# Risks of Leukemia in Various Industrial Groups in Korea: A Retrospective National Cohort Study

**DOI:** 10.3390/ijerph20021187

**Published:** 2023-01-09

**Authors:** Won-Tae Lee, Woo-Ri Lee, Wanhyung Lee, Jin-Ha Yoon, Jongin Lee

**Affiliations:** 1 Department of Occupational and Environmental Medicine, Severance Hospital, Yonsei University College of Medicine, Seoul 03722, Republic of Korea; 2 Department of Public Health, Graduate School, Yonsei University, Seoul 03722, Republic of Korea; 3 Division of Cancer Control and Policy, National Cancer Control Institute, National Cancer Center, Goyang 10408, Republic of Korea; 4 Department of Occupational and Environmental Medicine, Gil Medical Center, Gachon University College of Medicine, Incheon 21565, Republic of Korea; 5 Department of Preventive Medicine, Yonsei University College of Medicine, Seoul 03722, Republic of Korea; 6 Department of Occupational and Environmental Medicine, Seoul St. Mary’s Hospital, The Catholic University of Korea College of Medicine, Seoul 06591, Republic of Korea

**Keywords:** leukemia, industry, occupational risk, standardized incidence ratio

## Abstract

It is known that occupational exposure to specific agents is associated with leukemia. However, whether the occupational risks of leukemia differ among various industrial groups remains unclear. Therefore, the purpose of this study was to elucidate the occupational risks of leukemia among different worker groups by industry. Data for a total of 11,050,398 people from the National Health Insurance System’s claim data from 2007 to 2015 were analyzed. By cohort inclusion of workers whose industry had not changed for three years and with total workers as a control group, the risk for a specific industry group was expressed as an age-standardized incidence ratio (SIR). Among groups by industry, ’Manufacture of motor vehicles and engines for motor vehicles’, ’Sale of motor vehicle parts and accessories’, and ’Personal care services’ showed significantly higher SIRs. In division analysis, the ’Manufacture of other machinery and equipment’ and ’Waste collection, treatment and disposal activities’ divisions showed significantly higher SIRs than other divisions. We identified an increased risk of leukemia in workers of certain industries in Korea. Based on the results of this study, it is necessary to create a policy to protect workers at risk of leukemia. Various additional studies are needed to protect workers by revealing more precise relationships between individual hazardous substances, processes, and leukemia.

## 1. Introduction

Leukemia is a hematologic malignancy characterized by the proliferation of white blood cells. Its carcinogenicity is well-known compared to other occupation-related cancers [1]. The International Agency for Research on Cancer (IARC) has listed various carcinogens associated with hematologic malignancies, including benzene, 1,3-butadiene, formaldehyde, and radiation exposure. Several occupations can lead to exposure to agents that could cause leukemia. There is sufficient evidence that benzene can cause acute myeloid leukemia (AML). Although it is usually desirable to assess the effects of a particular substance when assessing carcinogenic risk, most occupations involve simultaneous exposure to multiple chemicals [2]. Therefore, unlike experimental conditions in which all conditions can be controlled, epidemiological studies are sometimes conducted on the carcinogenicity of a defined specific occupational group. The IARC has also listed occupational groups such as the rubber production industry and occupations involving painting as risk factors for hematologic malignancy [3,4,5,6].

Working environment management has reduced exposure to carcinogens for many years [7]. Although exposure to carcinogens in large enterprises can be significantly reduced, it might be difficult to reduce similar exposure in relatively small workplaces. A recent study in China also pointed out that chronic benzene exposure continues in small and medium-sized workplaces [8]. Undetection causes biased assessment in occupational cancer. In case of large companies, they can register cancer patients by operating carcinogen reduction programs with financial support [9]. However, it is difficult to expect such programs from small companies.

The coexistence of carcinogen exposure and other risks makes carcinogenicity assessment more difficult. Competing risk can also be accepted as a kind of healthy worker effect. In other words, before the occurrence of leukemia, other fatal medical conditions could result from the same exposure to occupational hazards. For example, styrene is not known to be associated with lung cancer; however, it has been shown to present significant risk considering competing risks [10]. Considering this, it is estimated that the currently surfaced risk of disease occurrence due to occupational exposure is underestimated.

An increased risk of leukemia was identified in a case–control study conducted in the United States on agriculture and healthcare workers, janitors, cleaners, and light truck drivers [11]. Carpet installers, laborers, and plumbers were identified as those most affected by leukemia among construction workers [12]. However, most exploratory studies have been conducted with a case–control design. Studies using large-scale occupational data have not been reported yet. Therefore, this study aimed to identify industries with a high risk of leukemia among all Korean workers. 

## 2. Materials and Methods

### 2.1. Study Participants

This study used claims data from the National Health Insurance System (NHIS). In Korea, all medical activities are unified and claimed by the NHIS. To make a claim to the NHIS, a doctor must submit a claim stating the disease code and the name of the drug. Therefore, NHIS claim data contain information on medical use for the entire Korean population. Since this study aimed to identify risks by industry, workers whose industry classification was confirmed were included. A total of 11,050,398 workers were included. 

It is known that leukemia has a relatively short latency period compared to other tumors from occupational risks. A previous study even suggested that the risk becomes absent 10–15 years after exposure to benzene [13]. Additionally, even though there is an argument against this suggestion [14], we considered that a longer follow-up would draw bias because leukemia risk increases with age. Therefore, those aged 65 years of age or above were excluded from this study so as to include only the working population.

### 2.2. Diagnostic Definitions

We defined leukemia cases if the workers’ medical records contained a specific code for the disease issued by a physician. The three-digit 6th Korean Standard Classification of Diseases (KCD) diagnosis code was used for disease classification. The KCD is the Korean version of the ICD-10. Most of its disease codes are the same as the ICD-10. There was no major revision to the ICD-10 during the study period. Cases involving codes C91 (lymphoid leukemia), C92 (myeloid leukemia), C93 (monocytic leukemia), C94 (other leukemias of specified cell type), and C95 (leukemia of unspecified cell type) were defined as leukemia.

### 2.3. Industry Classification

Worker’s industries were classified according to Korean Standard Industry Classification (KSIC) by checking qualification information for each year. KSIC classifies industries into sections, divisions, groups, and classes with a top-down method. We analyzed the industries of 21 sections, 77 divisions, and 213 groups [15]. To exclude occurrences not related to occupational history of the target industry before working in it, we defined the washout period as one year from the start year of the cohort to the year in which the work history began. That means cases that occurred during this period were excluded from the analysis. Since meaningful information could be provided when there was sufficient exposure to unknown harmful factors in the target industry, subjects whose industry had not changed for three years, based on the base year for observation being 2009, were selected for the analysis. 

For example, ID 1 in Table 1 worked in the same industry (‘a’) from 2007 to 2015. In this case, the washout period was 2007 and the industry was classified as ‘a’. ID 6 worked in two industries but did not meet the minimum definition period. Thus, this case was not classified as belonging to a specific industry group and excluded in the analysis. Several examples are shown in Table 1 with this definition. 

### 2.4. Statistical Analysis

Leukemia cases were calculated according to sex, age, and industry group. Standardized incidence ratio (SIR) was calculated with the indirect standardization method. The reference group was all workers in Korea. The SIR for those aged from 25 to 64 years of age was calculated as the ratio of the actual number of cases to the calculated expected number of cases for each control group. The 95% confidence interval was estimated by the Poisson distribution. The top 10 industries with the highest lower limit of the 95% confidence interval among SIRs calculated among industry groups and divisions were presented after stratification by sex. Other industries are presented in Supplementary Tables. SAS version 9.4 (SAS Institute, Cary, NC, USA) and R version 4.2.0 (R Foundation for Statistical Computing, Vienna, Austria) were used for all statistical analyses. This study was approved by the Institutional Review Board (IRB) of Yonsei University (IRB number: Y-2017-0100). Data were anonymized prior to disclosure by the NHIS.

## 3. Results

Among 11,050,398 workers tracked from 2007 to 2015, 17,829 (0.16%) workers were diagnosed with leukemia. Among total workers, 66.6% were males and 33.4% were females. At the time of enrollment, 17.4% of the cohort belonged to the 35–39 years old age group. Young workers aged 25–44 years old were mainly included. The proportion of older workers was relatively low. The proportion of leukemia patients was 0.17% in men, slightly higher than that in women. By age group, the ratio showed a tendency to increase with age. The ratio was 0.32% for those aged 60 to 64 years old (Table 2).

In the results of industry groups, ‘Manufacture of motor vehicles and engines for motor vehicles’, ’Sale of motor vehicle parts and accessories’, and ’Personal care services’ showed significantly higher SIRs of 1.44 (95% CI 1.06–1.90), 1.98 (1.05–3.38), and 2.74 (1.01–5.97), respectively. High SIRs were also found for ’Building of ships and boats’ and ’Building construction’. However, their lower limits for 95% confidence intervals included values of 1. When stratified by sex, SIRs for ’Personal care services’, ’Manufacture of special-purpose machinery’, and ’Building of ships and boats’ were significantly higher in male workers. In women, significantly higher SIRs were found for those in ’Other human health activities’, ’Passenger land transport, except transport via railways’, ’Manufacture of other non-metallic mineral products’, and ’Manufacture of leather, luggage and similar products’ groups (Table 3). Results from other industrial groups are listed in Supplementary Table S1.

In division analysis, which was an upper classification of the group, significantly higher SIRs were found for the ‘Manufacture of other machinery and equipment’ and ‘Waste collection, treatment and disposal activities’ groups. The same results were found for male workers as well. In women, significantly higher SIRs were found for those in the ‘Land transport and transport via pipelines’, ‘Manufacture of other non-metallic mineral products’, and ‘Social work activities’ groups (Table 4). Results from other industrial divisions are listed in Supplementary Table S2. In the case of large-class section analysis, a section with a statistically significant increase in SIR was not identified.

## 4. Discussion

In this study, industries with a high risk of developing leukemia were explored. Several industry groups and divisions were found to have significantly higher incidences of leukemia compared to the entire population of workers. Industrial groups or divisions with significantly high SIRs and their suspected occupational risks are listed in Supplementary Table S3.

Among industry groups, ’Manufacture of motor vehicles and engines for motor vehicles’ and ’Sale of motor vehicle parts and accessories’ showed a high incidence of leukemia. This might be related to exposure to substances during work. In particular, the substance known to be most closely related to the induction of leukemia is benzene. A number of epidemiological studies have shown that benzene can significantly increase the risk of leukemia [16]. Benzene is known to cause leukemia by inducing bone marrow damage when it changes to metabolites such as benzene oxide in the body [17]. However, the distinct relationship between benzene and leukemia also hinders the search for other causative factors. Because the Korean Industrial Accident Compensation Insurance Act specifies benzene-associated leukemia (exposure level above 0.5 ppm) as a work-related disease for compensation, researchers on occupational health have tended to focus on benzene exposure. However, other causative agents should be addressed; in a study reported in the 1960s, some components of automobile engine oil additives showed carcinogenicity in experimental animal studies [18]. Regarding the reason why a significant risk of leukemia appeared not only in the ’Manufacture of motor vehicles and engines for motor vehicles’ group, but also in the ’Sale of motor vehicle parts and accessories’ group, it might be because the place where car maintenance is performed may have been registered as a sales business. 

The ’Personal care services’ group includes barbers and hair beauty businesses. There have been reports of several diseases, including leukemia, that occur among hairdressers [19]. In addition to hair dye, the building environment of the beauty salons in which hairdressers work has been identified as an influential factor for leukemia and pre-leukemia [20], though it is difficult to judge whether factors such as construction materials and smoking are significant in the current Korean beauty salon environment. A meta-analysis that reported significantly increased risk related to personal use of hair dyes suggests the possibility of an increased risk of leukemia [21]. It should be considered that more cases than those represented in our data may exist because most hairdressers in Korea are self-employed and are thus often not covered by workers’ compensation insurance.

Additionally, the risk of developing leukemia in the ‘Manufacture of other non-metallic mineral products’ and ‘Manufacture of leather, luggage and similar products’ groups was analyzed to be high among female workers. Perturbation through penetration and damage caused by various chemicals used in the production of these types of products can lead to mutations that are involved in excessive cell proliferation [22]. Hematopoietic stem cells and related cells can be very vulnerable to the mutagenic or toxic effects of chemicals during differentiation. Thus, it is necessary to pay attention to the harmful factors of these industries. 

The crude incidence rate of leukemia identified in this study was 0.16%, that is, 16 cases per 100,000 people for eight years. According to 2010 Korean statistics, which is the median year of the period during which this study was conducted, the incidence of leukemia per 100,000 people was found to be about 5 cases, and the number of new cases was 2684 [23]. Even if we applied a strict definition for a worker’s industry, the incidence was not higher than the national leukemia incidence. Because of this healthy worker effect [24], it is possible the SIRs in this study may underestimate the real risk. Furthermore, all the study participants were covered by social insurance. However, the occupational environments of uncovered workers whose are expected to be more inadequate than covered workers. Therefore, a limitation of this study is that these precarious workers were not included.

Since cases were defined using only KCD diagnostic codes in this study, patients who were not actually confirmed might have been included in this study. However, these directions are not always consistent. It is known that both overestimation and underestimation are possible [25]. On the other hand, it is known that the spectrum of other diagnostic codes that occur due to bone marrow dysfunction, such as aplastic anemia (D61) and MDS (D46), share the same pathophysiology as AML [26]. However, they were not included in this study. Further studies should be conducted that take into consideration other diagnoses suggesting bone marrow abnormalities in addition to leukemia. 

Smoking is known to be a significant risk factor for leukemia [27]. In this study, individual risk factors for diseases such as smoking history were not considered. Since smoking rates vary by industry and occupation [28], the possibility that the risk of leukemia was relatively high in industries with high smoking rates could not be excluded. Lastly, the high screening rate of health checkups in certain industries might have caused some distortion in the statistics. However, in this study, the diagnosis code identified by the National Health Insurance System was used. Thus, the possibility of misrepresentation was not high because malignant tumors were strictly reviewed.

## 5. Conclusions

We identified an increased risk of leukemia in the workers of certain industries in Korea. As we already know, occupational exposure is an important risk factor for leukemia. In addition, based on results of this study, it is necessary to create a policy to protect workers at risk of leukemia. Furthermore, in order to protect workers by revealing more precisely the relationship between individual hazardous substances, processes, and leukemia, various additional studies are needed, such as exposure assessments and experimental studies.

## Figures and Tables

**Table 1:** Examples of industry definitions for workers in the study cohort.

ID	Annual Codes of Industrial Classification									Washout Period	Industry Definition
	2007	2008	2009	2010	2011	2012	2013	2014	2015		
1	a	a	a	a	a	a	a	a	a	2007	A
2	NA	b	b	b	NA	NA	NA	NA	NA	2007–2008	B
3	c	c	c	c	d	d	d	d	d	2007	C
4	e	e	f	f	f	f	f	f	f	2007–2009	F
5	NA	NA	NA	NA	NA	NA	g	g	g	2007–2013	NA
6	h	h	i	i	h	h	h	i	i	2007–2011	NA
7	j	j	j	NA	NA	NA	NA	NA	NA	2007	J
8	k	k	k	l	l	l	l	l	l	2007	K

NA: not available; small letters (a, b, c, d, e, f, g, h, i, j, k, l): examples of jobs.

**Table 2:** General characteristics of the study population and the number of leukemia cases.

		Total Workers		Leukemia (C91–C95)	
Total		11,050,398		17,829	(0.16%)
Sex	Male	7,362,615	(66.6%)	12,668	(0.17%)
	Female	3,687,783	(33.4%)	5161	(0.14%)
Age	25–29	1,853,391	(16.8%)	2089	(0.11%)
	30–34	1,907,448	(17.3%)	2287	(0.12%)
	35–39	1,925,593	(17.4%)	2525	(0.13%)
	40–44	1,697,347	(15.4%)	2587	(0.15%)
	45–49	1,494,043	(13.5%)	3053	(0.20%)
	50–54	1,147,382	(10.4%)	2360	(0.21%)
	55–59	657,070	(5.9%)	1740	(0.26%)
	60–64	368,124	(3.3%)	1188	(0.32%)

**Table 3:** Top 10 priority KSIC groups with a high risk of leukemia as assessed by age-standardized incidence ratios (SIRs) and a 95% confidence interval (95% CI).

Priority	KSIC Code	Classification of Industry	Expected	Observed	SIR (95% CI)
**Both sex**					
1	C301	Manufacture of motor vehicles and engines for motor vehicles	34	49	**1.44 (1.06–1.90)**
2	G452	Sale of motor vehicle parts and accessories	7	13	**1.98 (1.05–3.38)**
3	S961	Personal care services	2	6	**2.74 (1.01–5.97)**
4	C311	Building of ships and boats	25	35	1.43 (0.99–1.98)
5	F411	Building construction	64	79	1.23 (0.98–1.54)
6	Q869	Other human health activities	2	5	3.01 (0.98–7.02)
7	C291	Manufacture of general-purpose machinery	20	29	1.45 (0.97–2.09)
8	C292	Manufacture of special-purpose machinery	19	28	1.45 (0.96–2.09)
9	C339	Other manufacturing	228	247	1.08 (0.95–1.23)
10	H491	Transport via railways	10	16	1.65 (0.95–2.69)
**Male**					
1	S961	Personal care services	1	4	**4.14 (1.13–10.59)**
2	C292	Manufacture of special-purpose machinery	17	27	**1.55 (1.02–2.26)**
3	C311	Building of ships and boats	24	35	**1.43 (1.00–1.99)**
4	C301	Manufacture of motor vehicles and engines for motor vehicles	35	47	1.34 (0.98–1.78)
5	C291	Manufacture of general-purpose machinery	18	27	1.46 (0.96–2.12)
6	C339	Other manufacturing	192	204	1.06 (0.92–1.22)
7	G452	Sale of motor vehicle parts and accessories	6	11	1.82 (0.91–3.25)
8	L681	Real estate activities with owned or leased property	57	65	1.13 (0.88–1.45)
9	E381	Waste collection	1	4	3.19 (0.87–8.17)
10	F411	Building construction	61	68	1.12 (0.87–1.42)
**Female**					
1	Q869	Other human health activities	1	5	**7.34 (2.38–17.12)**
2	H492	Passenger land transport, except transport via railways	1	5	**4.97 (1.61–11.60)**
3	C239	Manufacture of other non-metallic mineral products	1	4	**5.41 (1.47–13.86)**
4	C151	Manufacture of leather, luggage and similar products	1	3	**5.42 (1.12–15.85)**
5	Q872	Non-residential welfare facilities	18	26	1.42 (0.92–2.07)
6	F411	Building construction	6	11	1.77 (0.88–3.16)
7	L681	Real estate activities with owned or leased property	14	20	1.42 (0.87–2.20)
8	G471	Retail sale in non-specialized stores	15	21	1.40 (0.86–2.13)
9	C243	Casting of metals	1	3	4.13 (0.85–12.07)
10	G461	Wholesale on a fee or contract basis	7	11	1.66 (0.83–2.97)

All results are expressed as SIR (95% CI). SIR: standardized incidence ratio; CI: confidence interval. Bolds—groups with statistical significance.

**Table 4:** Top 10 priority KSIC divisions with a high risk of leukemia as assessed by age-standardized incidence ratios (SIRs) and a 95% confidence interval (95% CI).

Priority	KSIC Code	Classification of Industry	Expected	Observed	SIR (95% CI)
**Both sex**					
1	C29	Manufacture of other machinery and equipment	39	57	**1.45 (1.10–1.88)**
2	E38	Waste collection, treatment and disposal activities, materials recovery	5	10	**2.15 (1.03–3.95)**
3	L68	Real estate activities	106	124	1.17 (0.98–1.40)
4	C33	Other manufacturing	229	249	1.09 (0.96–1.23)
5	F41	General construction	96	112	1.16 (0.96–1.40)
6	C31	Manufacture of other transport equipment	30	40	1.33 (0.95–1.80)
7	C30	Manufacture of motor vehicles, trailers and semitrailers	70	82	1.17 (0.93–1.45)
8	G45	Sale of motor vehicles and parts	9	15	1.65 (0.92–2.72)
9	H52	Warehousing and support activities for transportation	114	120	1.06 (0.88–1.26)
10	G47	Retail trade, except motor vehicles and motorcycles	112	112	1.00 (0.82–1.21)
**Male**					
1	C29	Manufacture of other machinery and equipment	36	54	**1.51 (1.13–1.96)**
2	E38	Waste collection, treatment and disposal activities, materials recovery	4	9	**2.18 (1.00–4.14)**
3	C31	Manufacture of other transport equipment	30	40	1.34 (0.96–1.83)
4	L68	Real estate activities	85	99	1.17 (0.95–1.42)
5	C30	Manufacture of motor vehicles, trailers and semitrailers	64	76	1.18 (0.93–1.48)
6	C33	Other manufacturing	192	206	1.07 (0.93–1.23)
7	F41	General construction	91	99	1.09 (0.88–1.32)
8	C18	Printing and reproduction of recorded media	16	22	1.39 (0.87–2.10)
9	H52	Warehousing and support activities for transportation	112	117	1.04 (0.86–1.25)
10	G45	Sale of motor vehicles and parts	8	13	1.57 (0.84–2.68)
**Female**					
1	H49	Land transport and transport via pipelines	2	8	**3.63 (1.57–7.15)**
2	C23	Manufacture of other non-metallic mineral products	2	5	**3.27 (1.06–7.63)**
3	Q87	Social work activities	23	34	**1.46 (1.01–2.04)**
4	G47	Retail trade, except motor vehicles and motorcycles	30	39	1.32 (0.94–1.80)
5	Q86	Human health activities	38	47	1.22 (0.90–1.63)
6	S96	Other personal services activities	10	15	1.55 (0.87–2.56)
7	L68	Real estate activities	21	25	1.21 (0.78–1.78)
8	C33	Other manufacturing	41	43	1.04 (0.76–1.41)
9	F41	General construction	10	13	1.35 (0.72–2.31)
10	G46	Wholesale trade on a personal account or on a fee or contract basis	24	26	1.08 (0.70–1.58)

All results are expressed as SIR (95% CI). SIR, standardized incidence ratio; CI, confidence interval. Bolds—groups with statistical significance.

## Data Availability

Data from NHIS can be accessed after permission: https://nhiss.nhis.or.kr/.

## Supplementary Materials

The following are available online at https://www.mdpi.com/article/10.3390/ijerph20021187/s1, Tables S1–S3. Supplementary Table S1: List of KSIC groups with a high risk of leukemia as assessed by age-standardized incidence ratios (SIRs) and a 95% confidence interval (95% CI). Supplementary Table S2: List of KSIC divisions with a high risk of leukemia as assessed by age-standardized inci-dence ratios (SIRs) and a 95% confidence interval (95% CI). Supplementary Table S3: Industries with a high risk of leukemia and suspected occupational factors.
